# Pattern analysis of schistosomiasis prevalence by exploring predictive modeling in Jiangling County, Hubei Province, P.R. China

**DOI:** 10.1186/s40249-017-0303-5

**Published:** 2017-04-26

**Authors:** Shang Xia, Jing-Bo Xue, Xia Zhang, He-Hua Hu, Eniola Michael Abe, David Rollinson, Robert Bergquist, Yibiao Zhou, Shi-Zhu Li, Xiao-Nong Zhou

**Affiliations:** 1 National Institute of Parasitic Diseases, Chinese Center for Disease Control and Prevention, Key Laboratory of Parasite and Vector Biology, Ministry of Health, WHO Collaborating Center for Tropical Diseases, Shanghai, 200025 People’s Republic of China; 2 Jiangling Institute of Schistosomiasis Control and Prevention, Jiangling, 434100 People’s Republic of China; 3 Department of Zoology, Natural History Museum, Wolfson Wellcome Biomedical Laboratories, Cromwell Road, London, SW7 5BD UK; 4 Ingerod, Brastad, Sweden; 5 Department of Epidemiology, School of Public Health, Fudan University, Shanghai, 200032 People’s Republic of China

**Keywords:** Schistosomiasis, Clustering, Predictive modelling

## Abstract

**Background:**

The prevalence of schistosomiasis remains a key public health issue in China. Jiangling County in Hubei Province is a typical lake and marshland endemic area. The pattern analysis of schistosomiasis prevalence in Jiangling County is of significant importance for promoting schistosomiasis surveillance and control in the similar endemic areas.

**Methods:**

The dataset was constructed based on the annual schistosomiasis surveillance as well the socio-economic data in Jiangling County covering the years from 2009 to 2013. A village clustering method modified from the K-mean algorithm was used to identify different types of endemic villages. For these identified village clusters, a matrix-based predictive model was developed by means of exploring the one-step backward temporal correlation inference algorithm aiming to estimate the predicative correlations of schistosomiasis prevalence among different years. Field sampling of faeces from domestic animals, as an indicator of potential schistosomiasis prevalence, was carried out and the results were used to validate the results of proposed models and methods.

**Results:**

The prevalence of schistosomiasis in Jiangling County declined year by year. The total of 198 endemic villages in Jiangling County can be divided into four clusters with reference to the 5 years’ occurrences of schistosomiasis in human, cattle and snail populations. For each identified village cluster, a predictive matrix was generated to characterize the relationships of schistosomiasis prevalence with the historic infection level as well as their associated impact factors. Furthermore, the results of sampling faeces from the front field agreed with the results of the identified clusters of endemic villages.

**Conclusion:**

The results of village clusters and the predictive matrix can be regard as the basis to conduct targeted measures for schistosomiasis surveillance and control. Furthermore, the proposed models and methods can be modified to investigate the schistosomiasis prevalence in other regions as well as be used for investigating other parasitic diseases.

**Electronic supplementary material:**

The online version of this article (doi:10.1186/s40249-017-0303-5) contains supplementary material, which is available to authorized users.

## Multilingual abstract

Please see Additional file 1 for translation of the abstract into the five working languages of the United Nations.

## Background

Schistosomiasis causes serious harm to residents’ health and impedes economic development in endemic areas in China [1–4]. Since the implementation of National Middle- and Long-term Plan of Schistosomiasis Prevention and Control, remarkable progress has taken place with an overall downward trend of endemicity and prevalence in terms of schistosomiasis patients, infected animals and snails. As of 2015, among 12 schistosomiasis endemic provinces in China, five have reached the stage of transmission-interruption, namely, Shanghai, Zhejiang, Fujian, Guangdong, and Guangxi, while 7 other provinces, namely, Hunan, Hubei, Jiangxi, Anhui, Jiangsu, Sichuan and Yunnan have reached the stage of transmission control [5]. Although great achievement has been made in the past several decades, the risk of schistosomiasis still exists, especially in the lake and marshland areas, due to the suitable environment for intermediate snails’ development, frequent human and livestock activities [6–10]. Therefore, it is urgent to promote advanced studies for schistosomiasis surveillance and control, especially in the lake and marshland areas with low prevalence of schistosomiasis [11].

Data mining methods together with computational modelling has been playing an important role in the studies of schistosomiasis and has been applied widely in guiding field practice and designing epidemiology surveys. It is particularly useful to health planners and decision makers. A linear regression model found that the prevalence of schistosomiasis showed a significant linear regression relationship with ecological environmental factors including the riparian water table, annual rainfall and yearly evaporation and altitude in the endemic areas following the Three Gorges Construction [12]. In a further step, multivariate regression found that eliminating water contact in the month of July would reduce the prevalence of schistosomiasis in the population [13]. However, it is difficult to assess the risk factors of schistosomiasis that are believed to be non-linear by conventional statistical methods. Artificial neural network was found to be more suitable to be applied with the logistic model to illustrate the complex and nonlinear relationship between the risk rankings in schistosomiasis prevalence. The main risk factors of human infection with *Schistosoma japonicum* were people aged ≤15, people with lower education, residents in villages with higher infection rates, people belonging to a poor family and in populations where infections occurred often [14].

Recently, spatial-temporal cluster analysis has been widely used for schistosomiasis risk surveillance and timely response and to help prioritize intervention strategies and implementation targets. However, few reports were found using the matrix model integrated with spatial-temporal cluster analysis for the schistosomiasis surveillance. In this study, the pattern of schistosomiasis prevalence in the selected areas, which were located in lake and marshland regions, was analysed using cluster analysis methods and a matrix-based prediction model. The aim was not only to further develop appropriate surveillance strategies for the source of infection of schistosomiasis and its related factors, but also to provide a feasible scientific basis for the interruption of schistosomiasis.

## Methods

### Study site and data collection

**Fig. 1:**
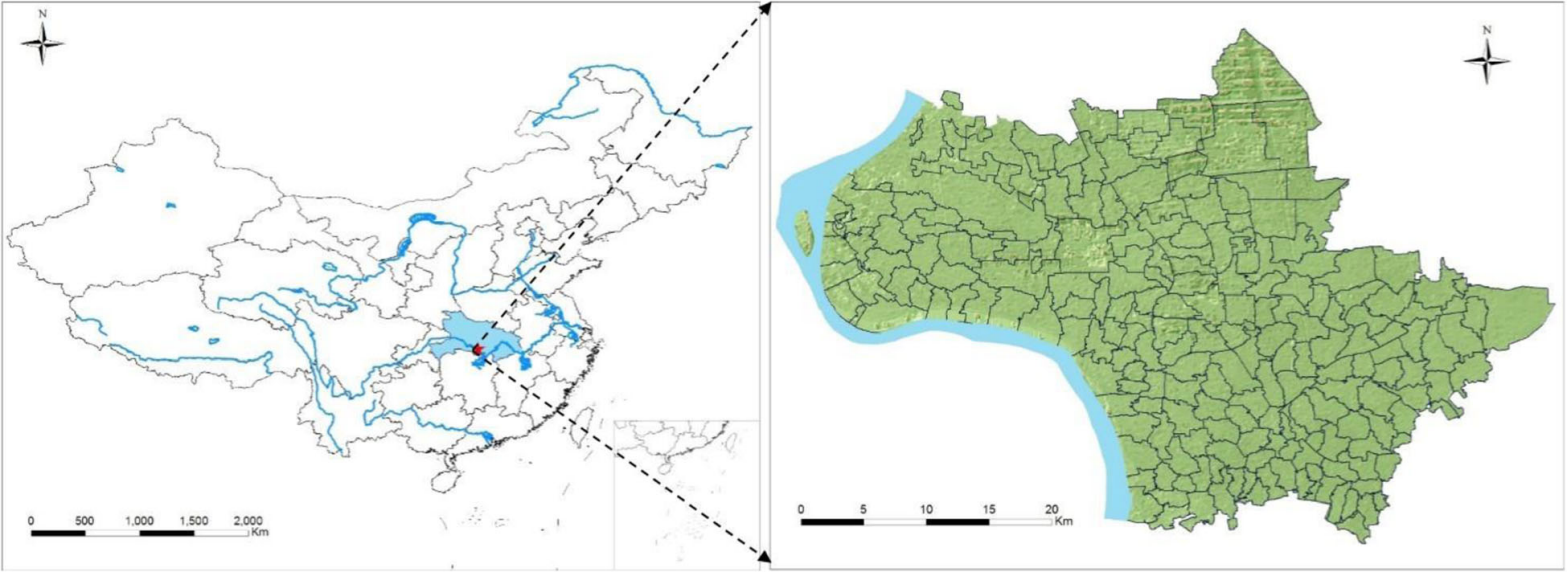
The map of study site, Jiangling County in southern Hubei Province, P.R. China

Jiangling County is a rural county of Hubei Province and located at the middle reaches of Yangtze River, which is known as a typical lake and marshland endemic area of schistosomiasis in China (Fig. 1) [15]. The dataset was constructed from the schistosomiasis annual surveillance program in Jiangling County and covered the total of 198 endemic villages during the years of 2009 to 2013. The collected data include the occurrence of schistosomiasis in human, cattle and snail populations [11]. In addition to the surveillance data, the social and economic indicators relating to endemic villages were collected and integrated into the dataset, which included the areas of water with and without infected snails, the number of cattle herds, the geographical area and the population size of each village.

### Village clustering analysis

In this study, clustering algorithm was explored to detect the different patterns of schistosomiasis prevalence in Jiangling County by means of data mining the temporal and spatial records of schistosomiasis occurrence in the 198 endemic villages. In doing so, the K-means clustering algorithm was applied and further modified by integrating the geographical locations of each village. The proposed clustering method parties *n* villages into *K* clusters in which each village belongs to the cluster with the nearest mean [16–18]. Given a set of villages (*x*_1_, *x*_2_, ⋯*x*_*n*_), where each village is a d-dimensional attributes, i.e., vector *x*_*i*_ = (*a*_*i*1_, *a*_*i*2_, ⋯*a*_*id*_). In this study, *x*_i_ represented a schistosomiasis endemic village, and *a*_*ij*_ denoted human schistosomiasis occurrence in village *i* and year *j*. The K-means clustering aims to partition the *n* observations into *k*(≤n) sets S = {*S*_1_, *S*_2_, ⋯ *S*_*k*_) so as to minimize the within-cluster sum of squares (WCSS). In other words, its objective is to find:

4 $$ \mathrm{argmin}{{\displaystyle {\sum}_{i=1}^k{\displaystyle {\sum}_{x\in Si}\left\Vert X-{\mu}_i\right\Vert}}}^2 $$

where *μ*_*i*_ is the mean of points in *S*_*i*_.

Silhouette plot was used to evaluate the cluster indices output from K-means algorithm in order to evaluate the performance of the resulting clusters. Silhouette values approaching 1 indicates that points are very distant from neighbouring clusters. By contrast, values below 0 and approaching -1 mean that points are not distinctly in one cluster or another. For each cluster *S*_*i*_, let *a*(*i*) be the average dissimilarity of *S*_*i*_, with all other data within the same cluster. Any measure of dissimilarity can be used but distance measures are the most common. It is then interpreted *a*(*i*) with regard to how well *S*_*i*_ is assigned to its cluster (the smaller the value, the better the assignment). Then it defined the average dissimilarity of point *b*(*i*) to the cluster *S*_*i*_ as the average of the distance from *S*_*i*_ to points in other clusters. The silhouette value for the *i* ^*th*^ point, *S*_*i*_, is defined as:

5 $$ {s}_i=\frac{b(i)- a(i)}{max\left\{ a(i), b(i)\right\}} $$

### Temporal predictive analysis

Predictive modelling exploits patterns found in historical schistosomiasis occurrence to identify infection risks and the correlations with its impact factors. In this study, the vector **Y** was used to represent the situation of schistosomiasis occurrences in three host populations, i.e., human, cattle and snail.

6 $$ \boldsymbol{Y}={\left({Y}_h,{Y}_c,{Y}_s\right)}^T $$

The vector **X** was utilized to denote five potential impact factors, including the water area (*X*_1_), the infected snail area (*X*_2_), the number of cattle herds (*X*_3_), village’s geographic area (*X*_4_) and the village population size (*X*_5_).

7 $$ \boldsymbol{X}={\left({X}_1,{X}_2,{X}_3,{X}_4,{X}_5\right)}^T $$

In epidemiology, the reproduction number *R*_0_ refers to the number of newly infection cases caused by a typical infectious individual in a completely susceptible population [19, 20]. Based on the compartmental models of schistosomiasis prevalence in humans, cattle and snails populations, the *R*( ⋅ ) denotes the temporal predictive function by the format of the reproduction number *R*_0_. *R*( ⋅ ) can be further determined by the impact factor vector **X**(t), that is ***R***(⋅) = ***R***(**X**(t)). In this regard, the dynamics of schistosomiasis prevalence can be estimated by the occurrences of schistosomiasis in the three mentioned host populations based on the last years schistosomiasis occurrence **Y**(t − 1) as well as the environment **X**(t) [21, 22], i.e. the impact factor vector **X**(t). Here, t denotes the current year.

8 $$ \boldsymbol{Y}\left(\boldsymbol{t}\right)=\boldsymbol{R}\left(\boldsymbol{X}\left(\boldsymbol{t}\right)\right)\cdot \boldsymbol{Y}\left(\boldsymbol{t}-1\right) $$

Furthermore, given that the occurrence rates of schistosomiasis in humans, cattle and snails populations in Jiangling County are near zero, a linearized assumption about the predictive function ***R***(**X**(t)) was used when **Y**(t) is close to zero.

9 $$ \boldsymbol{R}\left(\boldsymbol{X}(t)\right)=\boldsymbol{W}\cdot \boldsymbol{X}(t) $$

Therefore, matrix **W** can be interpreted as the temporal predication matrix

10 $$ \boldsymbol{W}=\left(\begin{array}{ccc}\hfill {w}_{h1}\hfill & \hfill \cdot \cdot \cdot \hfill & \hfill {w}_{h5}\hfill \\ {}\hfill {w}_{c1}\hfill & \hfill \cdot \cdot \cdot \hfill & \hfill {w}_{c5}\hfill \\ {}\hfill {w}_{s1}\hfill & \hfill \cdot \cdot \cdot \hfill & \hfill {w}_{s5}\hfill \end{array}\right) $$

while the way to estimate the parameters of matrix **W** is to compute the maximum likelihood estimation [23], which is defined as follows:

11 $$ \overset{\frown }{\mathbf{W}}\triangleq \arg\ \max\ \log\ p\left(\mathbf{\Re}\Big|\mathbf{W}\right) $$

### Validation by sampling faeces from domestic animals

In order to validate the results of spatial clustering and temporal prediction, this study carried out a faeces survey programme by covering different clusters of endemic villages. Schistosomiasis miracidia in the faeces samples was tested using the nylon hatching method. The samples were observed for at least 2 min at various times of incubation; the first, third and fifth hour for bovine and sheep faeces with the fifth and eighth hour for pig faeces. Positive faeces samples were also subjected to quantitative detection with the results recorded based on the presence and number of hatched miracidia observed with interpretation done by the single-blind method. Then, the results of local infection rates based on domestic animals’ faeces will be projected into the identified village clusters to validate the results of spatial clustering and temporal prediction.

## Results

### General status

**Fig. 2:**
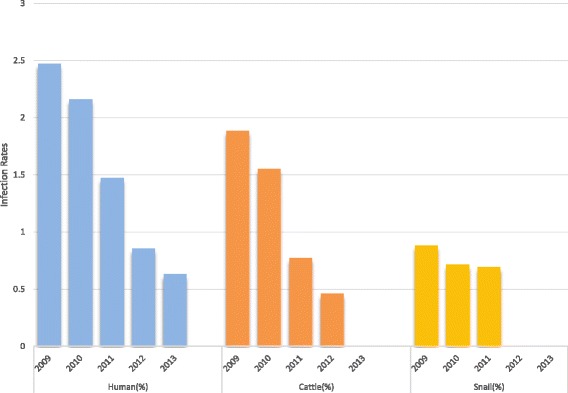
Schistosomiasis prevalence in Jiangling County during 2009 to 2013

The data of schistosomiasis annual surveillance among 2009 to 2013 showed that schistosomiasis prevalence in Jiangling County decline year by year (see Fig. 2). Specifically, the occurrence rate in humans decreased to 0.63% from the peak of 2.47% in 2009; that in cattle fell to zero in 2013 from 1.89% in 2009; while that in the snail populations were reduced to zero in 2012 from 0.88% in 2009.

### Villages clusters

**Fig. 3:**
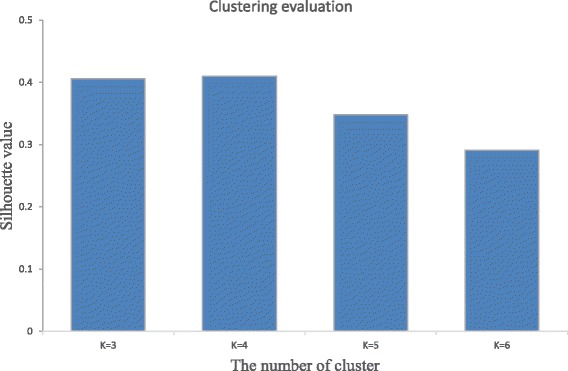
Silhouette function values for different number of spatial clusters

The K-mean clustering algorithm was applied to the 5 years’ dataset of schistosomiasis occurrence in human, cattle and snail populations. The number of expected clusters, i.e. the value of the parameter K, was established as 3, 4, 5 and 6, respectively. In order to determine the best number of clusters, the silhouette function is used to validate the results of spatial clustering in terms of the different number of generated clusters. As shown in Fig. 3, the best number of village clusters in this study was identified as four as it had the largest silhouette function value.

**Fig. 4:**
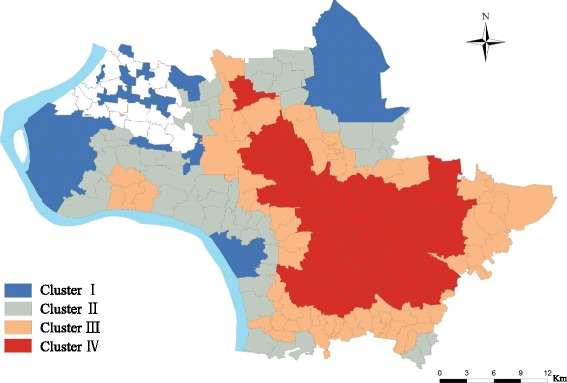
Spatial clusters with reference to schistosomiasis occurrence in humans from the year of 2009 to 2013

**Fig. 5:**
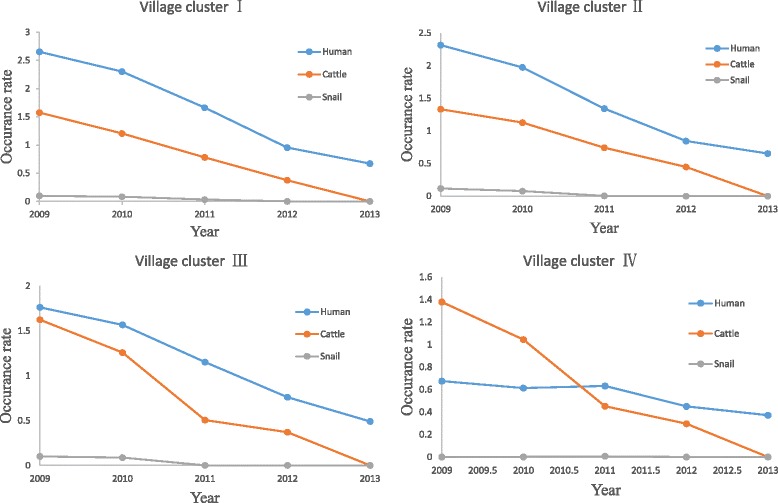
The pattern of schistosomiasis prevalence in the identified village clusters

The results of spatial clustering analysis showed that the total of 198 villages were categorized into four clusters with reference to the prevalence of schistosomiasis in three host population within the 5 years from 2009 to 2013. The geographical locations for these village clusters are depicted in Fig. 4. There were 71 villages categorized into Cluster I, 61 villages into Cluster II, 45 villages and 30 villages into Cluster III and Cluster IV, respectively. The patterns of schistosomiasis prevalence in each of the village clusters are demonstrated in Fig. 5. Within each of the identified village clusters, the prevalence patters are different from each other. Specifically, the Clusters I and II have relatively higher rates of schistosomiasis occurrence in human and cattle populations at the year of 2009. By the year of 2013, these two occurrence rates decreased remarkably synchronously. The Cluster III had a medium level of schistosomiasis prevalence in the year of 2009, and arrived at a similar level of schistosomiasis occurrence as these villages in Cluster I and II. Finally, villages in Cluster IV had the lowest level of schistosomiasis prevalence, especially in terms of human infection. However, the schistosomiasis elimination progress in these villages was hold back from 2009 to 2013. In general, the schistosomiasis prevalence in Jiangling County as a whole decline year by year. While the specific situation in each village clusters are different. The clustering algorithm made a deep mining on these annual surveillance data and showed the variations of prevalence pattern in each of village cluster.

### Predictive matrix

**Fig. 6:**
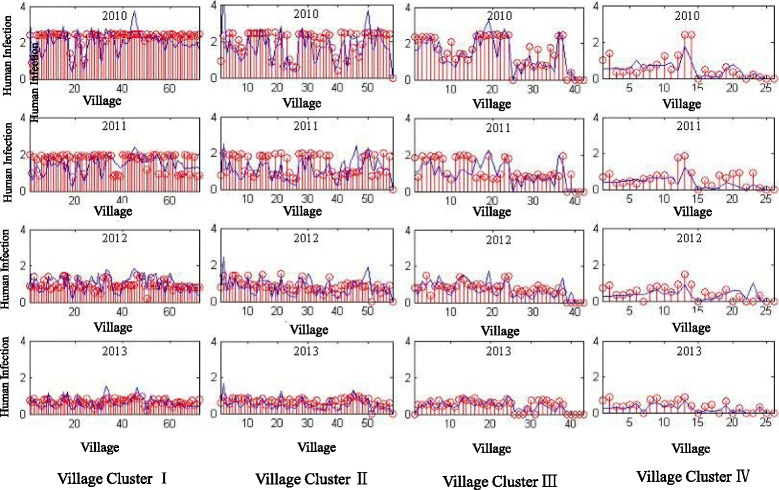
Temporal predictive analysis for schistosomiasis prevalence, in which the years from 2009 to 2012 are the training dataset and the year of 2013 the validation dataset

**Fig. 7:**
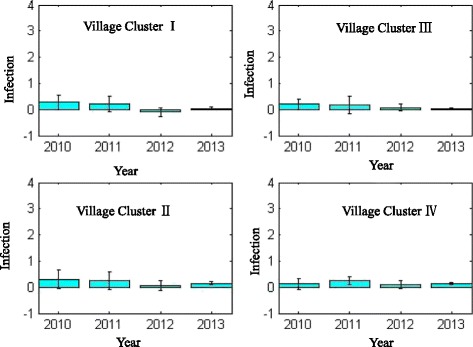
Predication error for each of the estimated prediction matrix

Based on the results of identified village clusters, it is assumed that the patterns of schistosomiasis prevalence can be further examined by exploring the predictive matrix, which revealed the associations between social-economic factors and schistosomiasis occurrence in humans, cattle and snail populations. It is used that the years from 2009 to 2012 as the training dataset and the year of 2013 as the validation dataset. The performance for each of the calculated predictive matrix is demonstrated in Fig. 6 with the predication error shown in Fig. 7. Based on the elements’ values in the predictive matrix of each village cluster, it is examined that the effects of five impact factors on the schistosomiasis prevalence. In terms of schistosomiasis prevalence in the human population, the population density, i.e., the population size and village’s geographical area, categorized the prevalence patterns in Clusters I and II, which means in the villages with a relatively higher level of schistosomiasis prevalence, the probability of human’s infectious exposure matters most. While the ratio between the infected snail area and the village geographical areas determine the attributes of village Cluster III and IV. This tells that in the less severe endemic villages, the effectiveness of vector control plays a key role in preventing schistosomiasis.

### Faeces survey

According to the results of clustered villages, the survey of faeces from domestic animals was conducted in environment with snails in the sampled villages selected from each of the village clusters. 701 faeces samples collected from six on-site surveys included faeces samples from 581 bovines, 112 sheep, 7 dogs and 1 pig with the proportions being 82.88%, 15.98%, 1.00% and 0.14%, respectively. The annual distribution of faeces was mainly in January, March and May when a total of 520 samples were collected, accounting for 74.18% of the total. The average distribution density was 0.0556/100 m^2^ and the biggest two locations were Jinma Village and Jinggan Village, namely, 0.6278 /100 m^2^ and 0.4019 /100 m^2^. The laboratory testing detected 82 positive faecal samples. The total infection rate of these positive samples was 11.70%, including faeces from 76 bovines and 6 sheep accounting for 92.68% and 7.32%, respectively. As illustrated in Table 1, the highest local rates of infection based on faeces from domestic animals were 30% (Lixin Village) and 19% (Xingxing Village). Then, the local infection rates based on domestic animals’ faeces will be projected into the identified village clusters. The results showed that villages in Cluster I have the highest level of infection from domestic animals’ faeces, while that of the villages in Cluster VI was closed to zero. These results helped to validate the results of spatial clustering.

**Table 1:** The surveyed rates of wild faeces infection in each selected sample villages

Cluster	Village	Positive rate(%)
Cluster I	Lixin	30
	Xingxing	19
	Gushu	15
Cluster II	Liukou	6
	Qiyuan	6
	Tancaidou	6
	Dengzhaogang	4
Cluster III	Jinggan	3
	Jinguosi	2
	Jinma	1
Cluster VI	Baiyang	0
	Yangyuan	0

## Discussion

The prevalence of schistosomiasis in China has been identified as a public health concern with a higher priority. The decades’ efforts has led to remarkable progress on the control and prevention of this disease [9, 24]. However, in current stage of lower infection rate the potential risks of direction infection still exist in certain regions, especially in the marshland and/or lake regions [25]. The source of infection is mainly livestock as the same distribution as that found in humans, including rebound human infection along increasing numbers of infected cattle. Finally, the snail host populations have increased significantly in the marshland and lakes regions in the endemic areas [5]. It is therefore important to identify the types of risks in different regions, so as to improve the capacity in schistosomiasis surveillance and control. Hubei Province is known as a hotspot of schistosomiasis, which can be attributed to the varying geographic landscapes of the entire region and the interplays among humans, cattle and snail populations, which are important components in the schistosomiasis surveillance framework [26]. The selected study site, Jiangling County located in the middle reaches of Yangtze River in Hubei Province, is one of the typical marshland and lake endemic areas of schistosomiasis [24, 27]. The National Schistosomiasis Surveillance Programme has been carried out in Jiangling County for several decades, and thus provides a data foundation with temporal and spatial records of infection cases that facilitates the understanding the operational situation in a low- prevalence endemic area.

In this study, two data mining methods in name of village clustering and prevalence prediction have been proposed to investigate the hidden patterns of schistosomiasis prevalence in such a marshland and lake endemic county. Existing spatial analysis methods, like the spatial autocorrelation analysis or hotspot analysis can find the geographical attributes of disease occurrence annually, but they fail with regard to determining the difference of disease occurrences in different years due to the effect of transmission [28]. It is found that this could be achieved by means of modifying the K-mean algorithm to identify village clusters from the historical records of schistosomiasis prevalence and the geographical locations of each village. The results provide a solution that divides these villages into different categories with reference to their temporal and spatial patterns of schistosomiasis prevalence.

In general, schistosomiasis prevalence in the study area declined year by year, while there was a differentiated trend when each village cluster was investigated, i.e. the villages in Clusters I and II demonstrated relatively more severe endemicity compared with the other three clusters. These analytical results agreed well with the real-world observations in the national schistosomiasis epidemic sampling survey [29, 30]. Based on the identified village clusters, it is further found that associated impact factors in the prevalence of schistosomiasis in human, cattle and snail populations using regression methods to explore the correlations between disease prevalence and its associated impact factors. As an extension of the conventional regression method, predictive matrix was applied by taking into account the complicated interplay between human, cattle and snail. In this way, the impact factors of schistosomiasis prevalence could be interpreted by last year occurrences in the three populations after adjusting the weights of each impact factors by the socioeconomic factors, including indicators of the areas of water with and without infected snails, the number of cattle herds, and the geographical areas of each village.

The generated predictive matrix in each village cluster can be used to characterize the difference of schistosomiasis prevalence in different regions, in which effects of each impact factors were different. These results agree with and also provide a solid foundation for integrated schistosomiasis control accordingly to the specific situations in each endemic region. The reliability of the predictive matrix method was validated both by a computational approach and the real-world survey-based validations, i.e. by comparing the simulated and the real schistosomiasis prevalence, the prediction errors were within the acceptable level. Furthermore, the results of the animal faeces investigations agreed with the potential schistosomiasis risks of each village cluster found.

## Conclusion

Due to the continuously efforts on control and prevention, schistosomiasis prevalence in Jiangling County is under a relatively low level. In this study, two research questions had been investigated: (1) how to differentiate the total of 198 endemic villages in Jiangling County with reference to their patterns of schistosomiasis prevalence temporally and spatially; (2) how to interpret and explain these identified differentiations. In order to answer these two questions, the methods of village clustering and prevalence prediction had been proposed and applied based on the collected dataset of schistosomiasis annual surveillance among the years of 2009 to 2013 in Jiangling County. The results of spatial clustering analysis in this study have shown that these endemic villages can be categorized four types of village cluster with reference to the temporal and spatial patterns of schistosomiasis prevalence. For each of the identified village cluster, the generated prediction matrix can be used to estimate next year schistosomiasis prevalence based on the current level of infection as well as their associated impact factors. The results of village clusters and the prediction matrix can be regard as the basis to conduct targeted measures for schistosomiasis surveillance and control. Furthermore, the proposed models and methods can be modified to investigate the schistosomiasis prevalence in other regions and even be used for other types of parasitic diseases.

## Additional file

Additional file 1: Multilingual abstract into five official working languages of the United Nations. (PDF 611 kb)
