# Supplementary material for: Pattern analysis of schistosomiasis prevalence by exploring predictive modeling in Jiangling County, Hubei Province, P.R. China
Source: Infect Dis Poverty. 2017 Apr 26;6:91. doi: 10.1186/s40249-017-0303-5 (PMC5406921; doi:10.1186/s40249-017-0303-5)
Supplement: Additional file 1: — Multilingual abstract into five official working languages of the United Nations. (PDF 611 kb) [file 40249_2017_303_MOESM1_ESM.pdf]

Translation of the abstract into the five official working languages of the United Nations

تحليل نمط انتشار مرض البلهارسيا في مقاطعة جيانغلين بمحافظة هوبي بجمهورية الصين الشعبية من خلال استكشاف النموذج التنبؤي

شانغ شيا، جينغ بو شيويه، شيا تشانغ، هيهوا هو، إنيولا مايكل أبي، ديفيد رولنسون، روبرت بيرجكويست، ييبياو تشو، شيجو لي، شياو نونغ تشو

الملخص

الخلفية: يعد إنتشار مرض البلهارسيا مشكلة صحية عامة رئيسية في الصين وتعتبر مقاطعة جيانغلين في محافظة هوبي بحيرة ومستنقع نموذجي موبوء بالبلهارسيا لذلك فإن تحليل نمط إنتشار البلهارسيا في مقاطعة جيانغلين له أهمية كبيرة وذلك لتحسين مراقبة ومكافحة المرض في المناطق الموبوءة المماثلة.

الأساليب: تم إنشاء قاعدة بيانات إستناداً إلى التقارير السنوية لمراقبة مرض البلهارسيا والبيانات الاجتماعية-الاقتصادية في مقاطعة جيانغلين عن الفترة من 2009 إلي 2013 وتم إستخدام إسلوب القرية التجميعي المعدل من متوسط خوارزمية كيعني للتعرف على الأنواع المختلفة من القرى الموبوءة وبعد ذلك تم إستخدام مجموعات القري التي تم تحديدها في وضع نموذج تنبؤي يعتمد على المصفوفة من خلال إستنتاج خوارزمية العلاقة الزمنية الرجعية بهدف تقدير الارتباطات الخطيرة لانتشار البلهارسيا بين السنوات المختلفة كما تم جمع عينات البراز من الحيوانات الداجنة وتم إستخدامها كمؤشر لاحتمالية انتشار البلهارسيا واستخدمت هذه النتائج للتحقق من صحة نتائج النماذج والأساليب المقترحة .

النتائج: تشير النتائج إلي انخفاض انتشار مرض البلهارسيا في مقاطعة جيانغلين المساهمة مع مرور السنين ويمكن تقسيم ال 198 قرية الموبوءة في مقاطعة جيانغلين إلي أربع مجموعات إستنادا إلي بيانات إصابة البلهارسيا في الإنسان والماشية والقواقع في الخمس سنوات محل الدراسة وتم إنشاء مصفوفة تنبؤية لوصف العلاقات بين إنتشار مرض البلهارسيا وبين مستوي الإصابة التاريخية بالمرض وعوامل التأثير المرتبطة بها وذلك لكل مجموعة من القرى التي تم تحديدها, وقد اتفقت نتائج عينات البراز التي تم تجميعها من الحقل مع نتائج المجموعات المحددة من القرى الموبوءة.

الخلاصة: تعتبر نتائج المجمعات القروية والمصفوفة التنبؤية التي تم إستخدامها في الدراسة الحالية كأساس الذي من خلاله يتم إجراء التدابير المستهدفة لمراقبة مرض البلهارسيا ومكافحته وعلاوة على ذلك فإنه من الممكن تعديل النماذج والأساليب المقترحة لإستخدامها في التحقيق في انتشار مرض البلهارسيا في مناطق أخرى وكذلك للتحقيق في أمراض طفيلية أخري غير البلهارسيا.

Translated from English version into Arabic by Mohamed R. Habib

基于预测模型的中国湖北江陵地区血吸虫病流行模式分析

夏尚, 薛靖波, 张瑕,胡合华, Eniola Michael Abe, David Rollinson, Robert Bergquist, 周艺彪, 李石柱, 周晓农

摘要:

引言: 血吸虫病依然是中国公共卫生安全的重大威胁。湖北省江陵县是典型的湖沼型血吸虫病流行区。分析研究江陵县血吸虫病流行模式对于在类似的湖沼流行区开展血吸虫病监测与防治工作具有重要意义。

方法: 收集2009-2013年江陵县的血吸虫病疫情资料以及当地社会经济发展统计数据，采用基于K平均算法的聚类方法对疫区内的村进行分类，基于分类结果构建矩阵预测模型，通过采用一阶时间逆序关联推演算法分析血吸虫病在不同年份之间的关联关系。最后采用现场野粪调查数据作为风险指标验证模型和方法。

结果: 江陵县血吸虫病疫情呈现出逐年下降的趋势。根据2009-2013年血吸虫病人群、耕牛和钉螺感染率分布可以将疫区198个村分为四类，针对每一个村聚类，算法生成的预测矩阵可以刻画血吸虫病在不同年份之间的流行趋势及其与环境影响因素之间的关系。现场野粪调查结果与村聚类结果相一致。

结论: 村聚类及预测矩阵的结果可以作为血吸虫病监测与控制在不同地区分类实施的基础。进而，研究中模型和方法通过进一步延伸和拓展，以用于研究血吸虫病在其他地区或者其他重要寄生虫病的流行模式。

Translated from English version into Chinese by Shang Xia

Analyse de tendances de prévalence de la schistosomiase en étudiant la modélisation prédictive dans le comté de Jiangling, Province de Hubei, R.P. Chine

Shang Xia, Jing-Bo Xue, Xia Zhang, He-Hua Hu, Eniola Michael Abe, David Rollinson, Robert Bergquist, Yibiao Zhou, Shi-Zhu Li, Xiao-Nong Zhou

Résumé

Contexte: La prévalence de la schistosomiase demeure un important problème de santé publique en Chine. Le comté de Jiangling dans la province de Hubei est à la fois une zone typicalement lacustre et une zone marecageuse endémique. L'analyse de tendances de prévalence de la schistosomiase dans le comté de Jiangling est d'importance majeure afin de promouvoir la surveillance et la lutte contre la schistosomiase dans des zones endémiques similaires.
Méthodes: L'ensemble de données a été construit sur la base de la surveillance annuelle de la schistosomiase aussi bien que les données socio-économiques dans le comté de Jiangling couvrant la période allant de 2009 à 2013. Une méthode de regroupement de village modifiée de l'algorithme k-mean a été utilisée pour identifier les différents types de villages endémiques. Pour ces groupes de villages, un modèle prédictif à base de matrice a été mis au point au moyen d'explorer l'algorithme d'interférence de corrélation temporelle visant à estimer les corrélations prédictives de la prévalence de la schistosomiase au cours de différentes années. L'échantillonnage de fèces d'animaux domestiques, comme un indicateur de prévalence potentielle de la schistosomiase a été effectué et les résultats ont été utilisés pour valider les résultats des modèles et méthodes proposés.
Resultats: La prévalence de la schistosomiase dans le comté de Jiangling a diminué d'année en année. Le nombre total de 198 villages endémiques dans le comté de Jiangling peut être divisé en quatre groupes en référence aux cinq années de schistosomiase dans les population humaine, de bovins et de physes. Pour chaque groupe de village identifié, une matrice prévisionnelle a été générée afin de caractériser les relations de prévalence de la schistosomiase avec l'historique des taux d'infection ainsi que leurs facteurs d'impact associés. De plus, les résultats de l'échantillonnage des fèces dans les champs sont compatibles avec ceux des groupes de villages endémiques recensés.
Conclusion: Les résultats des groupes de villages et la matrice prédictive peuvent être tenus comme base pour effectuer des mesures ciblées de surveillance et de lutte contre la schistosomiase. En outre, les modèles et méthodes proposés peuvent être modifiés pour l'étude de la prévalence de la schistosomiase dans d'autres régions et être aussi utilisés pour étudier d'autres maladies parasitaires.

Translated from English version into French by Kokouvi Kassegne

Анализ образа по распространенности шистосомоза путём изучения прогностического моделирования в уезде Jiangling провинции Хубэй, КНР

Shang Xia, Jing-Bo Xue, Xia Zhang, He-Hua Hu, Eniola Michael Abe, David Rollinson, Robert Bergquist, Yibiao Zhou, Shi-Zhu Li, Xiao-Nong Zhou

Реферат

Фон: Распространенность шистосомоза остается ключевой проблемой общественного здравоохранения в Китае. Уезд jiangling в провинции Хубэй является типичной озерно-болотистой эндемичной зоной. Анализ образа по распространенности шистосомоза в уезде jiangling имеет важное значение для содействия наблюдения шистосомоза и управления в аналогичных эндемичных районах.
Методы: Набор данных был построен на основе годового наблюдения шистосомоза, а также социально-экономические данные в уезде Jiangling, охватывающие период с 2009 по 2013 год. Деревенский метод кластеризации изменен из алгоритм K-mean и был использован для определения различных видов эндемичных сел. Для этих выявленных деревенских кластеров,

прогностическая модель на основе матрицы была разработана с помощью того, что исследовать один-шагоый темпоральный вывод корреляционного алгоритма с целью оценки прогностической корреляции по распространенности шистосомоза среди разных лет. Полевой отбор проб фекалий от домашних животных, как показатель потенциальной распространенности шистосомоза, было проведено, и его результаты были использованы для проверки результатов предложенных моделей и методов.
Результаты: Распространенность шистосомоза в уезде Jiangling год в год снижается. В общей сложности 198 эндемичных деревень в уезде Jiangling можно разделить на четыре группы с учетом вхождения в 5 лет шистосомоза у человека, крупного рогатого скота и улиток. По каждому выявленному сельскому кластеру, прогностическая матрица создается для того, чтобы охарактеризовать отношения между распространенностью шистосомоза и историческим уровнем инфекции, а также между связанными факторами влияния. Кроме того, по результатам отбора проб фекалий от передней области согласился с результатами выявленных кластеров эндемичных сел.
Заключение: Результаты деревенских кластеров и прогностической матрицы можно рассматривать в качестве основы для проведения целенаправленных мер для наблюдения и контроля шистосомоза. Кроме того, предложенные модели и методы могут быть изменены, чтобы изучить распространенность шистосомоза в других регионах, а также быть использованы при исследовании других паразитарных заболеваний.

Translated from English version into Russian by Hao-Qi Zhang

Análisis del patrón de prevalencia de schistosomiasis mediante la exploración de un modelo predictivo en el Condado de Jiangling, Provincia de Hubei, P.R. China

Shang Xia, Jing-Bo Xue, Xia Zhang, He-Hua Hu, Eniola Michael Abe, David Rollinson, Robert Bergquist, Yibiao Zhou, Shi-Zhu Li, Xiao-Nong Zhou

Abstracto:

Trasfondo: La prevalencia de schistosomiasis sigue siendo uno de los mayores problemas de salud en China. El condado de Jiangling en la provincia de Hubei posee un lago típico y un área endémica de la marisma. El análisis del patrón de la prevalencia de Schistosomiasis en el condado de Jiangling es de importancia significativa para así promover la vigilancia y el control de schistosomiasis en áreas similarmente endémicas.
Métodos: El conjunto de datos fue construido basado en la vigilancia anual de Schistosomiasis, así como en los datos socioeconómicos del Condado de Jiangling para los años entre 2009 y 2013. Un método agrupador de proveniente del algoritmo K-promedio modificado fue utilizado para identificar distintos tipos de aldeas endémicas. Para estos grupos de aldeas identificadas, un modelo predictivo basado en matriz fue desarrollado mediante la exploración del algoritmo de un solo paso de inferencia y correlación temporal en reverso con el objetivo de estimar las correlaciones predicativas de la prevalencia de schistosomiasis durante diferentes años. Se llevó a cabo un muestreo de heces fecales de animales domésticos, como un indicador potencial de la prevalencia de schistosomiasis y los resultados fueron utilizados para validar los resultados obtenidos mediante los modelos y métodos propuestos.
Resultados: La prevalencia de schistosomiasis en el Condado de Jiangling ha declinado año tras año. Un total de 198 aldeas en el Condado de Jiangling pueden ser divididas en cuatro grupos en referencia a 5 años de ocurrencia de schistosomiasis en poblaciones de humanos, ganado y caracoles. Para cada grupo de aldeas identificado, una matriz predictiva fue generada para caracterizar la relación entre la prevalencia de schistosomiasis con el nivel de infección histórico, así como de sus factores de impacto asociados. Además, los resultados de las muestras de heces fecales obtenidas del campo concuerdan con los resultados de los grupos de aldeas endémicas identificadas.
Conclusión: Los resultados de estos grupos de aldeas y la matriz predictiva pueden ser considerados la base para conducir medidas específicas para la vigilancia y control de Schistosomiasis. Además, los modelos y métodos propuestos pueden ser modificados para investigar la prevalencia de Schistosomiasis en otras regiones, así como utilizados para la investigación de otras enfermedades parasíticas.

Translated from English version into Spanish by Laura C Vicente Rodriguez
